# A Comparison of Contrast-Enhanced Voiding Urosonography (CE-VUS) and Contrast Retrograde Cystourethrography (RCUG) for the Detection of Vesicoureteral Reflux in Young Dogs

**DOI:** 10.3390/ani15131918

**Published:** 2025-06-29

**Authors:** Georgia Trikoupi, Paraskevi Papadopoulou, Katerina K. Adamama-Moraitou, Maria Eleni Filippitzi, Savvas Deftereos, Christos Koutinas, Michail Patsikas, Frederica Papadopoulou

**Affiliations:** 1 Laboratory of Diagnostic Imaging, Department of Clinical Studies, School of Veterinary Medicine, Faculty of Health Sciences, Aristotle University of Thessaloniki, 54627 Thessaloniki, Greece; vivipap@vet.auth.gr (P.P.);; 2 Companion Animal Clinic, Department of Clinical Studies, School of Veterinary Medicine, Faculty of Health Sciences, Aristotle University of Thessaloniki, 54627 Thessaloniki, Greece; kadamama@vet.auth.gr (K.K.A.-M.);; 3 Laboratory of Animal Health Economics, School of Veterinary Medicine, Aristotle University of Thessaloniki, 54124 Thessaloniki, Greece; mefilippi@vet.auth.gr; 4 Department of Radiology and Diagnostic Imaging, School of Medicine, Democritus University of Thrace, 68100 Alexandroupolis, Greece; 5 Pediatric Ultrasound Center, 54624 Thessaloniki, Greece; fpapadopoulou@hotmail.com

**Keywords:** contrast-enhanced urosonography (CE-VUS), vesicoureteral reflux (VUR), radiographic retrograde cystourethrography (RCUG), dog, sensitivity, specific

## Abstract

The aim of this prospective study was to evaluate contrast-enhanced urosonography (CE-VUS) as an alternative to radiographic retrograde cystourethrography (RCUG) in the detection of vesicoureteral reflux (VUR) in young dogs. A total of 62 dogs, ranging in age from 6 weeks to 12 months, and 124 ureterorenal units (UUs) were investigated for VUR using RCUG and CE-VUS. After a baseline gray-scale ultrasound (US) of the urinary tract, a second-generation ultrasound contrast agent (UCA), diluted in a bottle of normal saline, was instilled into the urinary bladder via a catheter and CE-VUS was performed. VUR was detected when microbubbles were observed in the ureter and/or in the renal pelvis. In addition, RCUG was performed on the same day. The findings obtained by CE-VUS and RCUG were concordant in 117/124 UUs (94.35%). No reflux was detected in 101/124 UUs (81.45%) using both methods. With RCUG as the standard of reference, the sensitivity of CE-VUS was 94.12%, and the specificity was 94.39%. The positive predictive value was 72.73%, and the negative predictive value was 99.02%. CE-VUS is highly sensitive for the detection of VUR in young dogs.

## Simple Summary

This study evaluated the use of contrast-enhanced voiding urosonography (CE-VUS) as a radiation-free alternative to retrograde cystourethrography (RCUG) for detecting vesicoureteral reflux (VUR) in young dogs. While RCUG is the standard diagnostic method in veterinary medicine, CE-VUS has shown a high accuracy in pediatric human medicine. A total of 62 dogs aged 6 weeks to 12 months were examined using both CE-VUS and RCUG. This study assessed 124 ureterorenal units and found a 94.4% agreement between the two methods. CE-VUS demonstrated a 94.1% sensitivity and a 94.4% specificity, with a negative predictive value of 99%, indicating a strong reliability in ruling out VUR. Additionally, CE-VUS identified six cases of VUR not seen with RCUG. No adverse effects were reported, and CE-VUS was well tolerated. Its advantages include the absence of radiation, dynamic real-time imaging, and the ability to perform repeated evaluations. This study concludes that CE-VUS is a safe, accurate, and practical tool for diagnosing VUR in young dogs and may serve as a valuable alternative to RCUG in clinical practice.

## 1. Introduction

Vesicoureteral reflux (VUR) is defined as the retrograde regurgitation of the urine from the urinary bladder to the ureter upwards and into the collecting system of the kidneys [1,2,3,4]. The pathogenesis of primary VUR still remains unclear [5,6]. It seems that a possible mechanism for its development occurs at the vesicoureteral junction level [6,7,8]. By contrast, secondary VUR is associated with urinary bladder outlet obstruction, neurologic disease, and congenital anatomical or functional abnormalities at the ureterovesical junction, such as ectopic ureters [8,9,10].

VUR is the most common urinary tract abnormality in children [11,12]. It was first recorded in apparently normal children and infants in the 1950s to 1970s and prevalence was estimated to be from 0.4% to 1.8% [1,3]. According to the international literature, the incidence in dogs seems to be higher [4,10,13,14]. VUR was detected in 79% of puppies, 27% of adults and 10% of elderly dogs [13]. As described in a recent study the prevalence of VUR in 3-month-old puppies was 64–68% [4]. According to another study, the incidence of bilateral VUR was found to be 15% in healthy female dogs [14].

Contrast-enhanced voiding urosonography (CE-VUS) has been recognized as a safe, sensitive, and effective radiation-free imaging modality with a comparable or even higher sensitivity in comparison to retrograde cystourethrography (RCUG) for detecting and grading VUR and urethral imaging in pediatrics [11,12,15]. This method is performed with ultrasounds (USs), entailing urethral catheterization and the intravesical administration of an ultrasound contrast agent (UCA) [11,16]. CE-VUS has been incorporated into pediatric radiology and urology guidelines, and in many institutions it is used as a primary choice for diagnosis, replacing the conventional modalities of reflux imaging [1,11].

In dogs RCUG represents the only imaging technique for the detection of VUR [4,13,17,18]. This manuscript is a continuum of a recently published study, which assessed the feasibility of CE-VUS in the evaluation of the low urinary tract morphology and function in healthy dogs [19]. The aim of this study is to assess the diagnostic performance of CE-VUS, using second-generation UCA, in comparison to RCUG, for the diagnosis of VUR. Furthermore, this study aims to determine the incidence of VUR in young dogs. To the best of our knowledge, this is the first study investigating vesicoureteral reflux using CE-VUS in young dogs.

## 2. Materials and Methods

### 2.1. Animals

Client-owned young dogs, ranging in age from 6 weeks to 12 months, underwent CE-VUS with SonoVue^®^ (Bracco Imaging, Milan, Italy). for VUR diagnosis at the Laboratory of Diagnostic Imaging, Department of Clinical Studies, School of Veterinary Medicine, Faculty of Health Sciences, Aristotle University of Thessaloniki, Greece, from 2021 to 2023. Dogs were presented to our institution for health screening or diagnostic investigation of various diseases and underwent CE-VUS and RCUG for the detection of VUR.

The animal study protocol was approved by the Ethical Committee of School of Veterinary Medicine, Aristotle University of Thessaloniki (certificate no.: 757/23.05.2023) and by the State Veterinary Authorities (certificate no.: 254460(1176)/12.04.2023). Written consent was obtained from all the legal guardians of the dogs, after providing detailed information about the technique and the purpose of the study.

Finally, in our study we included only dogs with complete medical records and long-term follow-up data. Clinical data included medical history, physical examination findings, hematological analysis, and urinalysis. Urine culture was performed only if urinary tract infection was suspected due to the concurrent disease and/or urinalysis findings.

### 2.2. Procedure—Dog Preparation

All dogs were fasted for at least 6 h before the examination. Conscious sedation was performed in uncooperative dogs using intramuscular (IM) dexmedetomidine hydrochloride (Dexdomitor, Orion Corporation, Espoo, Finland) at 200 mcg/m^2^. Scanning in all dogs was performed after the insertion of a 20 or 22 Gauge IV catheter (B/Braun Medical Inc, Hessen, Germany) in the cephalic vein to ensure safety. The ventrolateral portion of the abdomen and external genitalia in female and male dogs was gently clipped.

#### 2.2.1. Contrast-Enhanced Voiding Ultrasonography (CE-VUS)

The CE-VUS imaging technique comprises five steps: pre-contrast US of the abdomen, urethral catheterization, preparation of UCA, intravesical administration of UCA, and post-contrast US of the abdomen [11].

Both pre-contrast US and CE-VUS after intravesical administration of UCA were performed using the same ultrasound machine (MyLabTMX8Vet, ESAOTE SpA, Genova, Italy) equipped with microconvex transducer mc 3–11 (3–11 MHz) and Contrast Tuned Imaging Technology (CnTI) (ESAOTE SpA, Genova, Italy). During CE-VUS examination, the mechanical index (MI) was set from 0.08 to 0.10. Additionally, a single focal zone was used, which was placed deeper in posterior bladder wall or kidneys.

##### Pre-Contrast US of the Abdomen

Prior to contrast studies, abdominal baseline gray-scale US was performed for all the dogs in right lateral recumbency via transabdominal approach, providing essential information about the anatomy and morphology of the urinary tract. The transducer was placed immediately in front of the pubic brim perpendicular to the skin in the midline in female dogs and to the left side of the prepuce in male dogs. Two complementary transverse and longitudinal planes were used to fully assess the bladder and proximal urethra. Then, the transducer was placed in the left and right cranial abdomen alternatively, and sagittal, axial, and coronal views of the kidneys were obtained.

A special effort was made to use and document the same standard planes and magnification of the urinary bladder, urethra, and kidneys before and after the administration of UCA, to facilitate comparison of the images. This is particularly important in cases in which the reflux is not obvious. Technically, pre-contrast US also allows the examiner to determine the optimal window to visualize the kidneys during the subsequent CE-VUS examination and to optimize imaging settings before UCA administration with the contrast imaging mode in dual display.

##### Urethral Catheterization

Urethra was catheterized under aseptic conditions. External genitalia were clipped and scrubbed with chlorhexidine gluconate 0.04% solution (Hibitane 4% *w*/*v*; Cana S.A., Athens, Greece). A sterilized vaginal speculum and an illuminator were used to place a 6− to 10− F foley catheter (Jørgen Kruuse A/S, Langeskov, Denmark) in the urethra of female dogs. A 1.3–2.0 mm buster catheter (Jørgen Kruuse A/S) was used in male dogs, depending on dog size and anatomy. The distal portion (2–5 cm) of the catheter was coated with a sterilized lidocaine hydrochloride anesthetic gel (Xylocaine Jelly 2%; Astra Zeneca AB, Sodartalla, Södertälje, Sweden) to lubricate the urethra during the insertion, minimizing discomfort. The catheter was secured without tension to the dog’s skin using a latex-free, hypoallergenic adhesive tape. Following catheter placement, the bladder was emptied.

##### Preparation of UCA

The second-generation UCA employed was the sulfur hexafluoride echo-signal enhancer SonoVue^®^ (Bracco Imaging, Milan, Italy). SonoVue^®^ was handled according to the manufacturer’s specific instructions outlined in the packaging insert and reconstituted just before use (1 mL contains 8 μL [45 μg] sulfur hexafluoride in microbubbles).

##### Intravesical Administration of UCA

A plastic bottle of 250 mL normal saline solution (sodium chloride 0.9%; Demo SA, Athens, Greece) at room temperature was used. One milliliter of the reconstituted SonoVue^®^ was injected into the bottle of normal saline under aseptic conditions. The bottle was shaken gently to homogenize the contents. Then, it was connected to the catheter and was placed 30 cm above the dog’s bladder level. The UCA–saline solution was infused into the bladder by drip-infusion until the dropping was stopped or the voiding was started.

The bladder and kidneys were scanned during and after bladder filling and voiding. Scanning of the proximal urethra was performed before and immediately after the start of the spontaneous micturition while removing the catheter into the distal urethra. The same standard views of the urinary bladder, bladder neck, proximal urethra, and kidneys were obtained as pre-contrast US.

##### Post-Contrast US

The US examination continued after voiding in a similar manner as above, always scanning the right and left kidney alternatively. Post-contrast scans of the bladder, urethra, and kidneys were performed after voiding, while the bladder was empty. The entire examination was documented on digital video clips and still images stored on the hard disk of the ultrasound recorder.

#### 2.2.2. Contrast Retrograde Cystourethrography (RCUG)

RCUG was performed using a digital radiographic and fluoroscopic system (Axiom Icons R100, Siemens AG, Medical Solutions, Munich, Germany) immediately after the completion of CE-VUS through the same urinary catheter left in place and after emptying the urinary bladder. The radiographic positive contrast agent employed was Omnipaque^®^ (GE Healthcare A.E., Athens, Greece), which consisted of water-soluble iodinated contrast media, iohexol (300 mg of iodine per mL or 647 mg of iohexol/mL). Contrast–saline solution 15–20% *w***/***v* was prepared and then infused into the bladder by drip-infusion until the dropping was stopped or the voiding was started in the same way as with CE-VUS. Left lateral recumbent, ventrodorsal, and/or oblique radiographs were obtained. Standard films were taken before contrast initiation, at the beginning of the bladder filling, at mild-full bladder, at full bladder, during voiding, and at the end of voiding.

### 2.3. VUR Assessment

All examinations were performed and assessed at the time of initial evaluation by two investigators who were also responsible for dogs’ clinical management. During CE-VUS it is essential to obtain homogeneous strong contrast agent density of the bladder content. The presence of microbubbles in the ureter or renal pelvis, appearing as strong hyperechoic signals in CnTI mode, was considered diagnostic of VUR. During RCUG examination, VUR was recorded if the radio opaque medium reached the ureters and/or renal pelvis.

The findings of both CE-VUS and RCUG examinations were graded in a similar manner according to the International Reflux Study Committee classification for RCUG as described in human medicine [20]. VUR with CE-VUS is classified into five grades (I–V) according to the degree of filling and dilation of the ureter and upper urinary tract (Table 1).

In all cases, vital signs were assessed during the examination by an anesthesiologist, including measurements of the heart rate and respiratory rate. Body temperature was also monitored at the end of the examination and 1 h thereafter. During CE-VUS, the dogs were systematically inspected for any signs of localized skin and/or mucosal tissue reaction, any pathological discharge, as well as generalized hypersensitivity, and acute or late hypersensitivity reactions. Afterwards, guardians were instructed to closely observe the dogs for a month and immediately inform the physicians in case of any adverse event.

### 2.4. Statistical Analysis

Descriptive statistics were used to summarize dogs’ demographic characteristics. For the statistical analysis of the results, to determine if there is a statistically significant association between each factor variable and the CE-VUS result, a Fischer’s exact test was performed. The usual parameters of diagnostic tests (sensitivity, specificity, positive predictive value, and negative predictive value) were calculated using RCUG as the reference standard. The statistical software program SPSS (SPSS version 25.0; IBM Corp., Armonk, NY, USA) was used. Significance was declared as *p* ≤ 0.05.

## 3. Results

Sixty-two dogs met the criteria and finally were included in our study. The mean age of the dogs was 4.6 months (range from 6 weeks to 12 months) and weighed from 1.1 to 28 kg (median 8 kg). A wide variety of breeds were represented. There were 24/62 (38.7%) mixed breed dogs and 38/62 (61.3%) purebred dogs. Among these dogs, 34 out of the 62 were male (54.8%) and 28 were female (45.2%). The dogs were admitted to the Clinic for health checks (55/62, 88.7%) or for a second opinion diagnostic investigation (7/62, 11.3%).

All dogs, with a total of 124 ureterorenal units (UUs), underwent CE-VUS and RCUG with the intravesical administration of SonoVue^®^ and Omnipaque^®^, respectively. All examinations were well tolerated. CE-VUS and RCUG were successfully performed in both unsedated (36/62, 58.1%) and sedated (26/62, 41.9%) dogs. No complications or side effects of the intravesical application of the two contrast agents were observed.

The CE-VUS examination revealed that 13/62 dogs (21%) had VUR, including 4/13 cases (30.7%) of unilateral VUR and 9/13 cases (69.3%) of bilateral VUR. This was found in 22/124 UUs (17.7%) with reflux grade distributions as follows: grade I and V = 0, grade II = 20/22 (90%), grade III = 1/22 (5%), and grade IV = 1/22 (5%). VUR was detected in 10/62 dogs (16.1%) using RCUG. This was in 17/124 UUs (13.7%) graded as follows: grade I and V = 0, grade II = 15/17 (88.2%), grade III = 1/17 (6%), and grade IV = 1/17 (6%). The results of the CE-VUS and RCUG examinations according to the classification of reflux studies are given in Table 2.

In our study, three dogs were diagnosed with VUR secondary to the ectopic ureter with both CE-VUS and RCUG. This was in four UUs with reflux grade distributions as follows: grade II = 2/4 (50%), grade III = 1/4 (25%), and grade IV = 1/4 (25%). In these cases, the results of the CE-VUS were in accordance with the results of the RCUG (Table 2).

**Figure 1:**
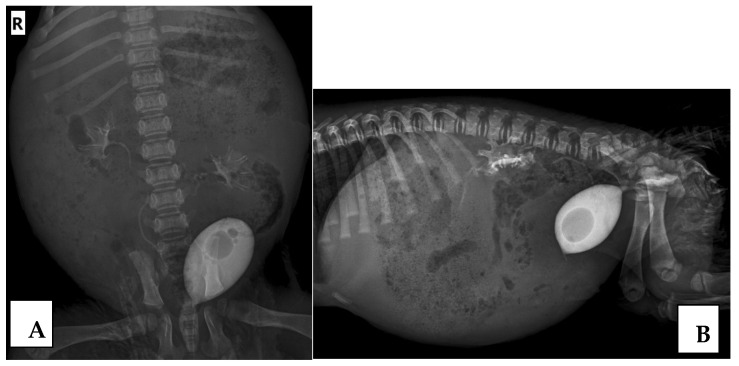
Vesicoureteral reflux (VUR) grade II in both the left and right kidney demonstrated on the retrograde cystourethrography (RCUG). The ventrodorsal (**A**) and lateral (**B**) view of the RCUG in the same dog.

**Figure 2:**
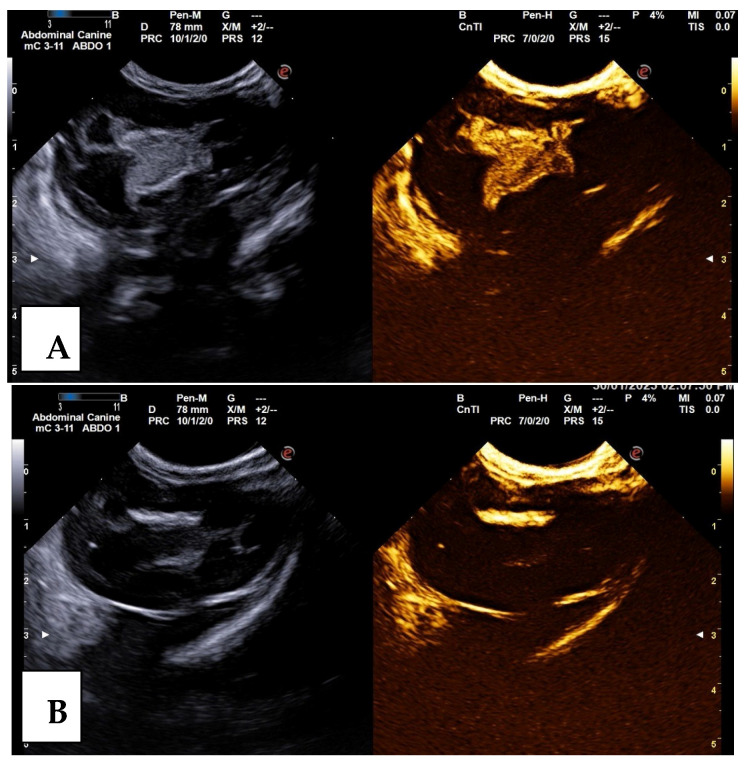
Vesicoureteral reflux (VUR) grade II in the left kidney demonstrated on the contrast-enhanced voiding urosonography (CE-VUS) in the dog presented in Figure 1. On CE-VUS microbubbles are visible in the renal pelvis (**A**), enabling the diagnosis of VUR. (**B**) The renal pelvis in the corresponding image before the contrast agent administration.

The results regarding the presence or absence of VUR by RCUG (Figure 1) and CE-VUS (Figure 2) were consistent in 117/124 UUs (94.4%). VUR on either method was found in 23/124 UUs (18.6%). VUR was detected with both imaging methods in 16/124 UUs (12.9%), by only CE-VUS in 6/124 UUs (4.8%), and by only RCUG in 1/124 UUs (0.8%). No reflux was detected in 101/124 UUs (81.4%) by both methods.

RCUG was used as the reference for the assessment of the sensitivity, specificity, and predictive values of CE-VUS. Using normal (no reflux) and abnormal (positive for reflux) results in the comparison of both methods, the sensitivity for CE-VUS was 94.12% (82.9–100%, 95%CI), and the specificity was 94.39% (90.0–98.8%, 95%CI). The positive predictive value was 72.73% (54.1–91.3%, 95%CI), and the negative predictive value was 99.02% (97.1–100%, 95%CI).

The median duration of the CE-VUS examination, without including the baseline sonography and catheter placement, was 17 min (range 10 to 40 min). The median duration of the RCUG was 23.9 min (range from 20 to 30 min).

The difference in the VUR detection with CE-VUS (CE-VUS− and CE-VUS+) between dogs of different ages and breeds was not found to be statistically significant. There were no significant differences found in the sedated status or pre-contrast US examination findings. However, statistically significant differences were noted between male and female dogs (*p* = 0.03) and between the dogs that presented to the Clinic for health checks and those presented for second opinion diagnostic investigations (*p* < 0.01) (Table 3).

The catheterization was uncomplicated and well tolerated by all dogs. No signs of acute hypersensitivity or anaphylactoid reactions were detected. No complications occurred during the examination or in the follow-up period. None of the dogs included in this study seemed to experience postprocedural adverse side events upon the telephone communication with and systematic questioning of the guardians one month after the examination.

## 4. Discussion

After a careful review of the veterinary literature of the last 50 years, only a few articles were found concerning reflux studies. All these articles were conducted with RCUG. To our knowledge this is the first study evaluating CE-VUS as an alternative modality of RCUG for the detection of VUR in young dogs. CE-VUS has been implemented for the diagnosis of VUR and other abnormalities of the lower urinary tract in medical practice, especially in children [11,12,16]. In veterinary medicine, CE-VUS has been described as a new, radiation-free alternative method for the imaging of the urinary bladder and urethra in healthy dogs [19,21].

VUR is the most common urological abnormality in children, ranging from 0.4% to 1.8% [1,2,3]. It is much more common in puppies and its incidence in dogs also varies from puppies to adults [4,13,22]. Our results are in discordance with those reported in a case series by Özkanlar et al., in which 64-68% of 3-month-old healthy puppies experienced VUR [4]. In the present study, VUR was found in 13/62 dogs (21%) with either CE-VUS or RCUG. This difference could be attributed to the different dog population that was under investigation. Age could be a significant factor since Özkanlar et al. evaluated very young dogs (3 months of age) [4]. It is well documented that the resolution of VUR may be seen upon maturity [22,23]. Furthermore, other factors that would be involved are the dissimilarities between the diseases of the dogs that were under investigation or the genetic background of the dogs examined in both studies.

There is no sex predisposition on the VUR presence in both humans and dog [5,22]. By contrast, in our study a statistically significant difference between male and female dogs was detected. Moreover, a statistically significant difference was found between the incidence of VUR in dogs presented for health checks and those presented for a second opinion diagnostic investigation (see Table 3). These results may be due to the inclusion of three female dogs with VUR secondary to the ectopic ureter—in our study, a fact that increases the number of VUR positive results in female dogs and in dogs presented for a second opinion diagnostic evaluations of their condition.

In human medicine, CE-VUS has become the primary screening method for children with suspected VUR in many places, with a high diagnostic accuracy [11,24,25,26]. CE-VUS has high diagnostic accuracy parameters (>90% sensitivity and specificity) and an increased positive diagnostic rate compared to RCUG [25,27,28,29,30]. In our study, the sensitivity and specificity were considered higher for CE-VUS using RCUG as the standard of reference.

In this study, CE-VUS detected six refluxes not detected by RCUG. The inherent nature of the UCA being composed of microbubbles in contrast to the X-ray contrast medium cannot be excluded as a possible cause for the difference between CE-VUS and RCUG results [24,25]. The chance of visualizing even a single microbubble aggregate by US makes it different from the X-ray contrast agent, where a certain concentration at a given time is needed to be detected [20,25].

Furthermore, VUR is a dynamic phenomenon and in up to 15–17% of cases is intermittent [11,13,25]. CE-VUS, as a radiation-free examination, enables the prolonged real-time observation of the entire filling and voiding process versus the shorter net fluoroscopy time, so CE-VUS has more chances to capture VUR than RCUG. On top of that, with CE-VUS we can record videoclips and images for the post hoc thorough assessment of the examination [11,16,31]. Consequently, the higher reflux detection rate by CE-VUS is attributed to the continuous and longer scanning times of the upper urinary tract instead of the intermittent radiography during the RCUG. The improved conspicuity of the ultrasound contrast is related to the newer generation of contrast and ultrasound techniques [25,29,32]. Moreover, according to a recent study, the ability of CE-VUS to diagnose more VUR than RCUG could be explained by the lower viscosity of the UCA that is almost identical to the urine viscosity [33]. Thus, during the CE-VUS procedure, the UCA might reproduce the urine’s movement in the urinary tract.

There was one case with VUR grade II detected with RCUG that was entirely missed on the CE-VUS. In this dog, VUR grade IV associated with an ectopic ureter was observed in the contralateral UU. The continuous scanning of the ectopic ureter resulted perhaps in the limited examination time of the opposite UU. The intermittent nature of VUR, especially in a low-grade reflux, could be the reason for this limitation [11,12].

In our study, all detected cases of VUR were classified as grades II–IV. This was probably related to the young age of the dogs. Dogs younger than twelve months of age develop an early secondary abnormality of the ureters and renal pelvis [4]. It was determined that the grade II VUR in dogs younger than 6 months old was related with the primary reflux, while grade III and IV VUR were diagnosed in dogs aged 6–12 months and were associated with the secondary reflux, including ectopic ureters. Transient primary VUR has a common prevalence and potential disappearance in time; thus, medical prevention should be recommended in dogs and children if an infection exists [4,11,23]. On the other hand, permanent secondary VUR must be managed surgically to prevent the development of severe abnormalities in kidneys and ureters [1,34].

VUR causes the retrograde flow of urine when there is an increase in bladder pressure [12,35]. This phenomenon occurs especially, but not exclusively, during the bladder voiding phase [16]. In the present study, all dogs could void around the catheter during CE-VUS, so the cyclic filling of the bladder and repeated CE-VUS examinations were feasible. This results in an increase in the VUR detection rate using CE-VUS.

After the last filling cycle, the catheter may be removed from the urinary bladder into the distal urethra during dog micturition, allowing further proximal urethra morphology and anatomy assessments [16,19,36]. This was important when urinary tract abnormalities were present in a dog, for instance as with ectopic ureters or urethral strictures [11]. In our study, this maneuver facilitated the visualization of the ureteral openings into the proximal portion of the urethra in dogs with VUR secondary to the ectopic ureter.

The contrast stability of a freshly prepared suspension of SonoVue^®^ was over 6 h. This implies that there is a potential for performing several studies from one vial that contains a 5 mL suspension [11,16]. Thus, the cost of the UCA, a major consideration for the widespread use of CE-VUS in veterinary clinical practice, could be reduced.

In the present study, no adverse events were reported during the procedure and the time after the CE-VUS examination. Previous studies in children reported that CE-VUS with the intravesical administration of SonoVue^®^ has an extremely high safety profile [11,12,15]. The few minor complications were attributed to the urethral catheterization [12,15]. Contrarily, RCUG is performed with the use of iodine contrast agents, which induce adverse reactions more frequently than ultrasound contrast agents [18,32]. In veterinary medicine, some minor adverse effects (i.e., vomiting) have been previously reported and were associated with the intravenous administration of SonoVue^®^ [37,38]. However, according to the literature, no adverse events have been reported after the intravesical administration of SonoVue^®^ in dogs [19,39].

In our study, we chose to perform the CE-VUS examination first and then the RCUG. This was done for two reasons. First, we did not want to be biased by knowing the results from the preceding RCUG. And second, this was according to the latest data published from studies conducted in children, comparing between CE-VUS and RCUG results, which were performed in the same manner as in our study [25,28,29,33]. In these studies, it is not reported that the CE-VUS procedure could have affected the urinary tract function or potentially impacted the results of the subsequent RCUG. Thus, we assert that performing the RCUG after the CE-VUS examination could not have influenced the evaluation of VUR [25,29].

The present study is limited by the small number of dogs included. Our results were based on 13 dogs with VUR out of 62 dogs checked in total. Additionally, in our population, given its low prevalence of a urinary tract pathology, we did not have the opportunity to study the entire spectrum of urinary tract abnormalities using the CE-VUS examination in comparison with RCUG. Further study in a larger population of dogs of varying urinary tract statuses is needed to confirm the results of this original research.

In conclusion, this study shows that CE-VUS has a high sensitivity for the detection of VUR. Therefore, CE-VUS may have the potential to reduce the use of RCUG, especially in follow-up investigations and postoperative examinations. The ability to perform cyclic filling without an extra radiation dose, the dynamic character of the examination in association with the dynamic nature of VUR, and the lack of risk associated with the administration of ionizing contrast agents appear to be the essential advantages of this modality. Additionally, the second-generation UCA proved to be durable in solutions for a long time, allowing enough time for the examination of the entire urinary tract. This enables the VUR screening in dogs to be more practical and permits repeated follow-up scans. Furthermore, a urethral assessment during the CE-VUS voiding phase is possible, providing its detailed anatomical and morphological imaging.

## 5. Conclusions

CE-VUS is a reliable examination modality providing a high specificity and accuracy in VUR diagnoses in dog populations. The image quality has proved to be equal or superior to the images received during the RCUG. In addition, there is no exposure to ionizing radiation for both the dog and the examiner. Finally, it is safe, relatively inexpensive, and can be conducted on a routine basis.

## Figures and Tables

**Table 1:** Vesicoureteral reflux (VUR) grading in contrast-enhanced voiding urosonography (CE-VUS) and retrograde cystourethrography (RCUG).

VUR Grading	
Grade I	Refluxing microbubbles in the ureter, which is not dilated.
Grade II	Refluxing microbubbles in the kidney collecting system, which is not dilated.
Grade III	Refluxing microbubbles fill the kidney collecting system with mild dilation and without change in its morphology.
Grade IV	Refluxing microbubbles fill the kidney collecting system, with moderate dilation and blunting of the renal fornices but with papillary impressions still visible.
Grade V	Refluxing microbubbles fill the kidney collecting system with marked dilation and loss of papillary impressions and tortuous course of the dilated ureter.

**Table 2:** Results of the contrast-enhanced voiding urosonography (CE-VUS) and retrograde cystourethrography (RCUG) examinations according to the classification of reflux studies in dogs with vesicoureteral reflux (VUR).

Dog	CE-VUS (Grade I–V)		RCUG (Grade I–V)	
	Right UU	Left UU	Right UU	Left UU
1	Grade II	Grade II	Grade II	Grade II
2	Grade II	No VUR	No VUR	No VUR
3	No VUR	Grade II	No VUR	Grade II
4	No VUR	Grade II	No VUR	Grade II
5	Grade II *	Grade III *	Grade II *	Grade III *
6	Grade II	Grade II *	Grade II	Grade II *
7	Grade II	Grade II	Grade II	Grade II
8	Grade II	Grade II	Grade II	Grade II
9	Grade II	Grade II	Grade II	No VUR
10	Grade II	Grade II	No VUR	No VUR
11	Grade II	Grade II	No VUR	No VUR
12	Grade II	Grade II	Grade II	Grade II
13	Grade IV *	No VUR	Grade IV *	Grade II

* In these ureterorenal units (UUs), VUR secondary to ectopic ureter was detected.

**Table 3:** Frequencies per variable (age, breed, sex, body weight, reason of presentation, pre-contrast US findings, and sedation) in 62 dogs with contrast-enhanced voiding urosonography (CE-VUS) positive (CE-VUS+, n = 13 dogs) and negative (CE-VUS−, n = 49 dogs) results for the detection of vesicoureteral reflux (VUR).

Variable		CE-VUS − n (%)	CE-VUS + n (%)	*p*
Age (months)	<6 months	26 (53%)	7 (54%)	1
	≥6 months	23 (47%)	6 (46%)	
Breed	Mixed	21 (43%)	3 (23%)	0.53
	Purebred	28 (57%)	10 (77%)	
Sex	Male	30 (61%)	4 (31%)	0.03
	Female	19 (39%)	9 (69%)	
Body weight	<5 kg	16 (33%)	5 (38,5%)	0.63
	≥5 kg to 10 kg	17 (34%)	5 (38,5%)	
	≥10 kg	16 (33%)	3 (23%)	
Reason of presentation	Health check	47 (96%)	8 (62%)	<0.01
	Second opinion diagnostic investigation	2 (4%)	5 (38%)	
US findings	No	43 (88%)	10 (77%)	0.19
	Yes	6 (12%)	3 (23%)	
Sedation	No	30 (61%)	6 (46%)	0.23
	Yes	19 (39%)	7 (54%)	

## Data Availability

Data presented in this study are available on request from the corresponding author.
